# During long days, HY5a keeps dormancy away

**DOI:** 10.1093/plcell/koae037

**Published:** 2024-02-06

**Authors:** Nora Flynn

**Affiliations:** Assistant Features Editor, The Plant Cell, American Society of Plant Biologists; Department of Botany and Plant Sciences, University of California, Riverside, CA 92521, USA

For us, short sleeves become winter coats to cope with unwelcoming conditions. But for perennial plants like poplar trees, the changing of seasons is weathered through careful timing of growth and dormancy. Photoreceptors, clock components, and effectors achieve a predictable cycle where active growth in the summer gives way to growth cessation and apical bud formation in the fall, protecting meristems through winter (Singh et al. 2017). **Yongfeng Gao and colleagues (**Gao et al. 2024**)** expose a new regulatory model for short-day–induced dormancy that involves a poplar (*Populus tomentosa*) ortholog of the Arabidopsis photomorphogenesis regulatory factor ELONGATED HYPOCOTYL 5 (HY5), HY5a. The model introduces poplar HY5a as a coordinator of growth cessation and bud set that promotes *FLOWERING LOCUS T2* (*FT2*) transcription through both direct and indirect routes (Fig).

**Figure.**
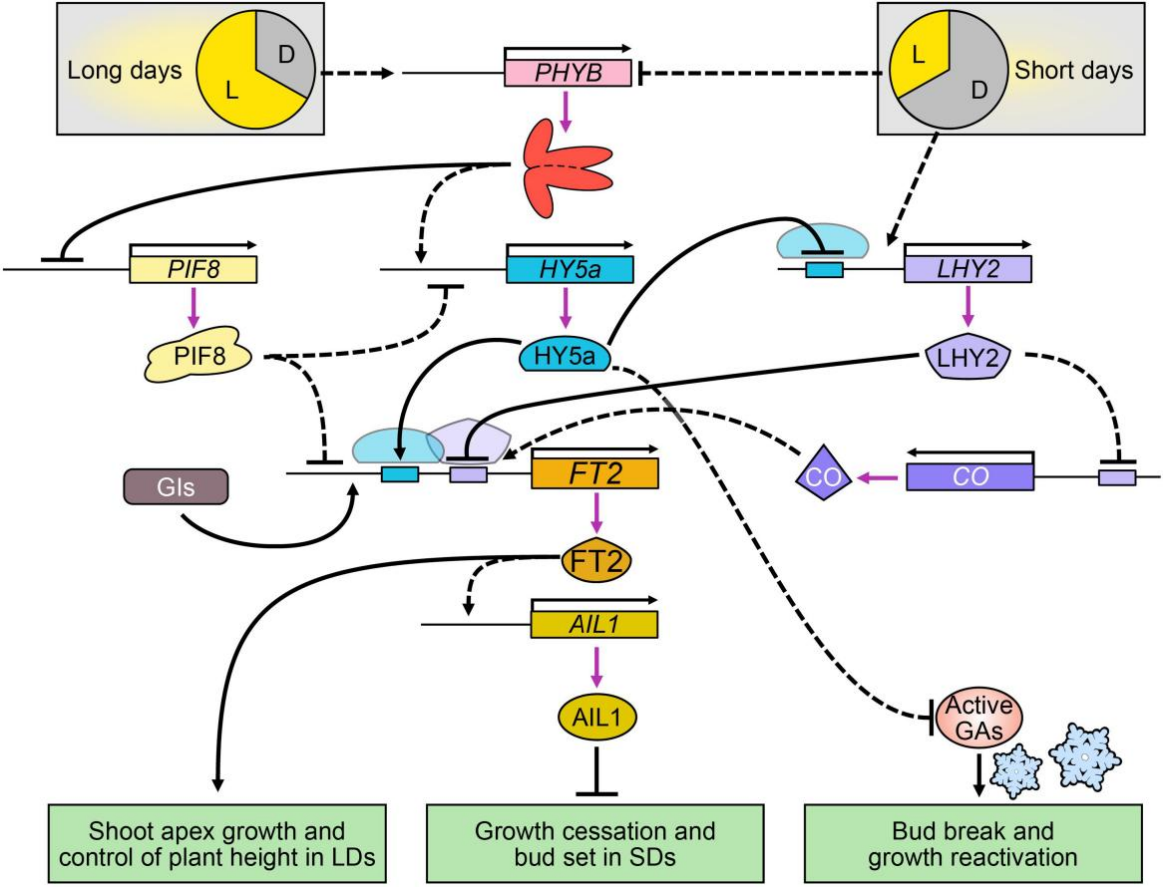
Model of PHYB2-HY5a-FT2 regulation during seasonal growth. Long photoperiods promote the expression of HY5a by activating PHYB, leading to increased FT2 expression, which promotes active growth in the apex through AIL1. Short days negatively regulate HY5a by inhibiting PHYB, contributing to growth cessation and bud set. Reprinted from Gao et al. (2024), Figure 8.

The authors first searched for uncharacterized transcriptional regulators of *FT2*, which is known to induce growth and delay dormancy during longer days (Singh et al. 2017). By using a yeast 1-hybrid assay with the *FT2* promoter as bait for a poplar cDNA library, they identified HY5a. Further analysis demonstrated that HY5a, a bZIP transcription factor, binds ACE-box (ACGT) motifs in the *FT2* promoter, enhancing *FT2* expression. Therefore, a novel HY5a-FT2 regulatory pathway may exist to temper dormancy induction.

To expose the intricacies of this new molecular pathway, the authors investigated if HY5a is linked to photoperiod-based seasonal regulation by using transgenic poplar overexpressing *HY5a* (*HY5a*-OE) and knockout lines (*HY5a*-KO). Whereas *HY5a*-KO plants began growth cessation, bud set, and bud break earlier than the wild type, *HY5a*-OE plants were late, suggesting that HY5a can suppress these seasonal changes. To tie together the impact of HY5a on the growth cycle with its regulation of *FT2*, the authors overexpressed *FT2* in *HY5a*-KO. The early growth cessation and bud set phenotypes of the *HY5a*-KO plants were suppressed by *FT2* overexpression, hinting that the premature responses of *HY5a*-KO are due to the lack of *FT2* activation by HY5a.

When comparing gene expression of *HY5a*-OE and *HY5a*-KO lines, the authors also noticed that the central clock component, *LATE ELONGATED HYPOCOTYL2* (*LHY2*), had lower expression when HY5a levels were high. *LHY* is known to be a target of HY5 in Arabidopsis (Lee et al. 2007) and was shown to inhibit *FT2* expression by binding to its promoter during short days, inducing growth cessation (Ramos-Sánchez et al. 2019). Therefore, the negative regulation of *LHY2* by HY5a may also indirectly increase *FT2* expression. Similar to *FT2*, *LHY2* contains ACE-box motifs, and HY5a could bind to these regions to inhibit its activity, revealing that whereas HY5a positively regulates *FT2* directly, it also negatively regulates *LHY2* to further stimulate *FT2* expression.

For HY5a to accurately regulate *FT2* as seasons change, it likely receives information on the photoperiod. The poplar phytochrome B orthologue (PHYB2) is involved in photoperiod perception and dormancy induction, but its method of action remains largely unknown. However, previous studies in Arabidopsis demonstrated that HY5 is involved in PHYB signaling (Lee et al. 2007). The authors found that PHYB2 overexpression leads to upregulation of *HY5a* and delayed growth. Loss of HY5a could negate this phenotype, indicating that HY5a is a downstream transcription factor of PHYB2.

Finally, the phenotypes of *HY5a*-OE and *HY5a*-KO plants indicated that *HY5a* may also be involved in bud burst. To further investigate this hypothesis, the authors compared the transcript abundances of genes related with the synthesis and breakdown of gibberellins (GA), plant hormones involved in dormancy release (Rinne et al. 2011). The activity of GA synthesis genes increased without HY5a, along with the levels of active GA. Therefore, *HY5a* is not only a novel regulator of short-day-induced dormancy in poplar but also a broader controller of seasonal growth that negatively regulates GA in buds.

## Dive Curated Terms

The following phenotypic, genotypic, and functional terms are of significance to the work described in this paper:

LHY Gramene: AT1G01060
LHY Araport: AT1G01060

PHYB Gramene: AT2G18790
PHYB Araport: AT2G18790

## References

[koae037-B1] Gao Y , Chen Z, Feng Q, Long T, Ding J, Shu P, Deng H, Yu P, Tan W, Liu S, et al ELONGATED HYPOCOTYL 5a modulates *FLOWERING LOCUS T2* and gibberellin levels to control dormancy and bud break in poplar. Plant Cell. 2024:36(5):1963–1984. 10.1093/plcell/koae022 PMC11062467 38271284

[koae037-B2] Lee J , He K, Stolc V, Lee H, Figueroa P, Gao Y, Tongprasit W, Zhao H, Lee I, Deng XW. Analysis of transcription factor HY5 genomic binding sites revealed its hierarchical role in light regulation of development. Plant Cell. 2007:19(3):731–749. 10.1105/tpc.106.047688 17337630 PMC1867377

[koae037-B3] Ramos-Sánchez JM , Triozzi PM, Alique D, Geng F, Gao M, Jaeger KE, Wigge PA, Allona I, Perales M. LHY2 integrates night-length information to determine timing of poplar photoperiodic growth. Curr Biol. 2019:29(14):2402–2406.e2404. 10.1016/j.cub.2019.06.003 31257141

[koae037-B4] Rinne PLH , Welling A, Vahala J, Ripel L, Ruonala R, Kangasjärvi J, van der Schoot C. Chilling of dormant buds hyperinduces FLOWERING LOCUS T and recruits GA-inducible 1,3-β-glucanases to reopen signal conduits and release dormancy in Populus. Plant Cell. 2011:23(1):130–146. 10.1105/tpc.110.081307 21282527 PMC3051240

[koae037-B5] Singh RK , Svystun T, AlDahmash B, Jönsson AM, Bhalerao RP. Photoperiod-and temperature-mediated control of phenology in trees—a molecular perspective. New Phytol. 2017:213(2):511–524. 10.1111/nph.14346 27901272

